# Association of apolipoprotein A1 with osteoporosis: a cross-sectional study

**DOI:** 10.1186/s12891-023-06264-6

**Published:** 2023-03-02

**Authors:** Xiaozhi Sun, Xiaotao Wu

**Affiliations:** 1 Department of Spine Surgery, Zhongda Hospital, School of Medicine, Southeast University, Nanjing, 210009 China; 2 Surgery Research Center, School of Medicine, Southeast University, 87# Dingjiaqiao Road, Nanjing, 210009 China

**Keywords:** Apolipoprotein A1, High-density lipoprotein cholesterol, Fracture, Bone mineral density, Osteoporosis

## Abstract

**Summary:**

Higher levels of apolipoprotein A1 (ApoA1) were associated with higher risk of osteoporosis, which supports the argument that lipid metabolism is involved in bone metabolism.

**Background:**

Although the current evidence shows that lipid metabolism and osteoporosis are closely related to cardiovascular disease, the association between ApoA1 and osteoporosis is still unknown. Therefore, the purpose of this study was to explore the relationship between ApoA1 and osteoporosis.

**Methods:**

In this cross-sectional study, we included 7743 participants in the Third National Health and Nutrition Examination Survey. ApoA1 was regarded as an exposure variable and osteoporosis was considered as an outcome variable. Multivariate logistic regression analysis, sensitivity analysis, and receiver operator characteristic (ROC) were used to assess the association of ApoA1 with osteoporosis.

**Results:**

Participants with higher ApoA1 had higher rates of osteoporosis compared to participants with lower ApoA1 (*P* <  0.05). Individuals with osteoporosis had higher levels of ApoA1 than individuals without osteoporosis (*P* <  0.05). In multivariate logistic regression analysis adjusted for age, sex, race, hypertension, diabetes, gout, hypotensive drugs, hypoglycemic drugs, systolic blood pressure, total cholesterol, low-density lipoprotein cholesterol, high-density lipoprotein cholesterol, apolipoprotein B, blood urea nitrogen, albumin, uric acid, hemoglobin A1c, alkaline phosphatase and total calcium, higher ApoA1 was strongly associated with higher risk of osteoporosis, whether as a continuous variable or a categorical variable [Model 3, OR (95% CI), *P* value: 2.289 (1.350, 3.881), 0.002 and 1.712 (1.183, 2.478), 0.004]. And after excluding individuals with gout, the correlation between them remained and was significant (*P* <  0.01). And ROC analysis also showed that ApoA1 could predict the development of osteoporosis (AUC = 0.650, *P* <  0.001).

**Conclusion:**

ApoA1 was closely associated with osteoporosis.

## Introduction

Osteoporosis is a chronic metabolic disease characterized by decreased bone mass, decreased bone mineral density, decreased bone strength and increased bone brittleness, resulting in a great increase in the risk of fracture [1]. There is evidence that almost half of Americans over the age of 50 are likely to suffer from fractures caused by osteoporosis [2]. Burge et al. showed that in 2005, approximately 2 million fractures occurred, costing up to $17 billion, with spinal fractures accounting for 27% of all fractures, and the annual fracture event and disease cost burden ars expected to rise by nearly 50% by 2025, suggesting that prevention of osteoporosis, not only in the hip but also in the spine, is very meaningful to reduce the risk of fracture [3]. Additionally, Odén et al. showed that more than 20 million men and nearly 140 million women worldwide were likely to suffer from fractures in 2010, with the highest number of fractures in both men and women in Asia, and the numbers are expected to double by 2040 [4]. In addition, not only fractures, but also severe osteoporosis can lead to arterial calcification and premature death and excess mortality [5–7]. And with the aging of the population, the prevalence of osteoporosis is on the rise, placing an enormous burden on health economies and health worldwide. It is clear that osteoporosis is not only a chronic metabolic bone disease, but also a serious public health problem, and therefore the identification of controllable risk factors for osteoporosis and early intervention are important and urgent to reduce the incidence and financial and health burden of osteoporosis-related diseases.

Current evidence suggests that age, postmenopausal women, vitamin D deficiency, poor nutrition, smoking, low calcium, glucocorticoid use, vitamin D deficiency, and a family history of fractures are inextricably linked to the development of osteoporosis [8–12], while the association of lipid metabolism with osteoporosis remains unclear. Apolipoprotein A1 is a low-regulated component of lipid metabolism, and it has been shown to have multiple cardiovascular protective effects as a major apolipoprotein of high-density lipoprotein cholesterol (HDL-C), however its role on bone metabolism remains unknown [13]. In a cross-sectional study involving 1791 participants, Wang et al. found that higher HDL-C levels were independently associated with a higher risk of osteoporotic fractures, so ApoA1, as the main carrier of HDL-C, may also be associated with osteoporosis, but the correlation between them has not been explored [14]. Therefore, to fill this knowledge gap, the present study aimed to explore the association of ApoA1 with osteoporosis in the general population in a large cross-sectional study.

## Materials and methods

### Study population

In this cross-sectional study, all participants were from the Third National Health and Nutrition.

**Fig. 1:**
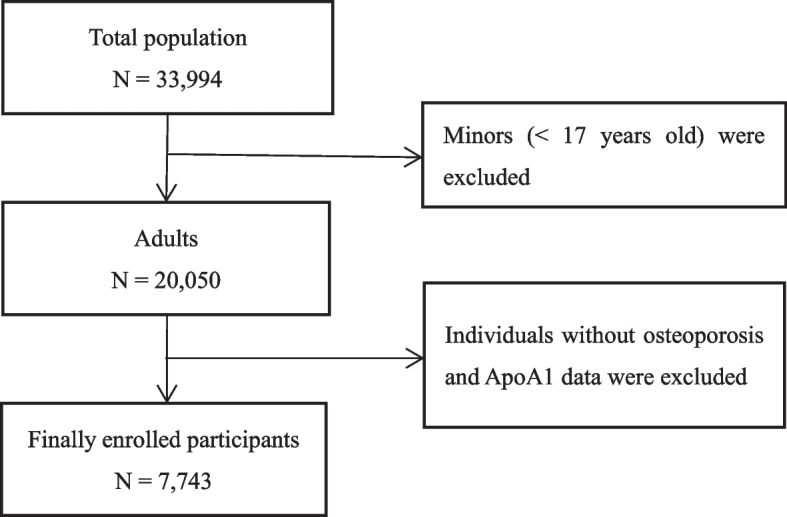
Flow chart of the study population. ApoA1, apolipoprotein A1

Examination Survey (NHANES III). After excluding minors (< 17 years old) and individuals without osteoporosis and ApoA1 data, 7743 individuals were included in the study for further analysis (Fig. 1). The study protocol regarding the NHANES III was approved by the National Center for Health Statistics of the Center for Disease Control and Prevention Institutional Review Board and adhered to the basic principles of the Declaration of Helsinki, and all individuals provided signed informed consent forms at the time of participation in NHANES III.

### Data collection and definitions

In this study, demographic variables included age, sex, race and smoking status. Among them, races were divided into four groups, namely, non-Hispanic White, non-Hispanic Black, Mexican-American and Others, while smoking status was divided into every day, some days and not at all. Comorbidities and medication covariates included hypertension, diabetes, hypercholesterolemia, gout, hypotensive drugs, hypoglycemic drugs and cholesterol-lowering drugs, in which hypertension was defined as the self-reported history of hypertension or being on oral hypoglycemic drugs or mean of three measurements of systolic blood pressure (SBP) / diastolic blood pressure (DBP) ≥ 140/90 mmHg. Diabetes was defined as a self-reported history of diabetes or being using hypoglycemic drugs or fasting plasma glucose (FPG) ≥ 7.1 mmol/L or hemoglobin A1c (HbA1c) ≥ 6.5% or blood glucose ≥11.1 mmol/L 2 hours after oral glucose tolerance test. Hypercholesterolemia was defined as self-reported history of hypercholesterolemia or being on oral cholesterol-lowering drugs. Gout was defined as a self-reported history of gout. Biomarker variables included body mass index (BMI), SBP, DBP, triglycerides (TG), total cholesterol (TC), low-density lipoprotein cholesterol (LDL-C), HDL-C, ApoA1, apolipoprotein B (ApoB), blood urea nitrogen (BUN), creatinine (CR), albumin, uric acid, FPG, HbA1c, C-reactive protein (CRP), alkaline phosphatase (ALP), total calcium and 25-hydroxyvitamin D [25(OH)D], where BMI was defined as the weight in kg divided by the square of the height in m, and these biomarkers were measured by trained professionals according to standard measurement procedures, the details of which were available on the NHANES official website. In our study, osteoporosis was defined as a self-reported history of osteoporosis or current use of anti-osteoporosis medication.

### Statistical analysis

In this study, continuous variables were expressed as mean ± standard deviation if they conformed to the normal distribution, while comparisons of differences between groups were made using independent samples T-test, otherwise they were expressed as median (interquartile range), while comparisons of differences between groups were made using nonparametric tests. Categorical variables were expressed as frequencies (percentages), and comparisons of differences between groups were performed using the Chi-square test or Fisher’s exact test. Univariate logistic regression analysis was then used to assess the association of each variable with osteoporosis and to select variables with *P* <  0.05 for the construction of adjusted models for multivariate logistic regression analysis. Model 1 included age, sex, and ApoA1; model 2 included age, sex, race, hypertension, diabetes, gout, hypotensive drugs, hypoglycemic drugs, and ApoA1; model 3 included the variables in model 2 as well as SBP, TC, LDL-C, HDL-C, ApoB, BUN, albumin, uric acid, HbA1c, ALP, total calcium and ApoA1. Then, we excluded individuals with gout for sensitivity analysis. And we used receiver operator characteristic (ROC) to assess the diagnostic value of ApoA1 for osteoporosis and to determine the optimal cutoff point of ApoA1. All statistical tests were performed using SPSS 26.0 and MedCalc 19.6.1, and a two-tailed *P* value < 0.05 was considered to be statistically significant.

## Results

### Baseline characteristics

**Fig. 2:**
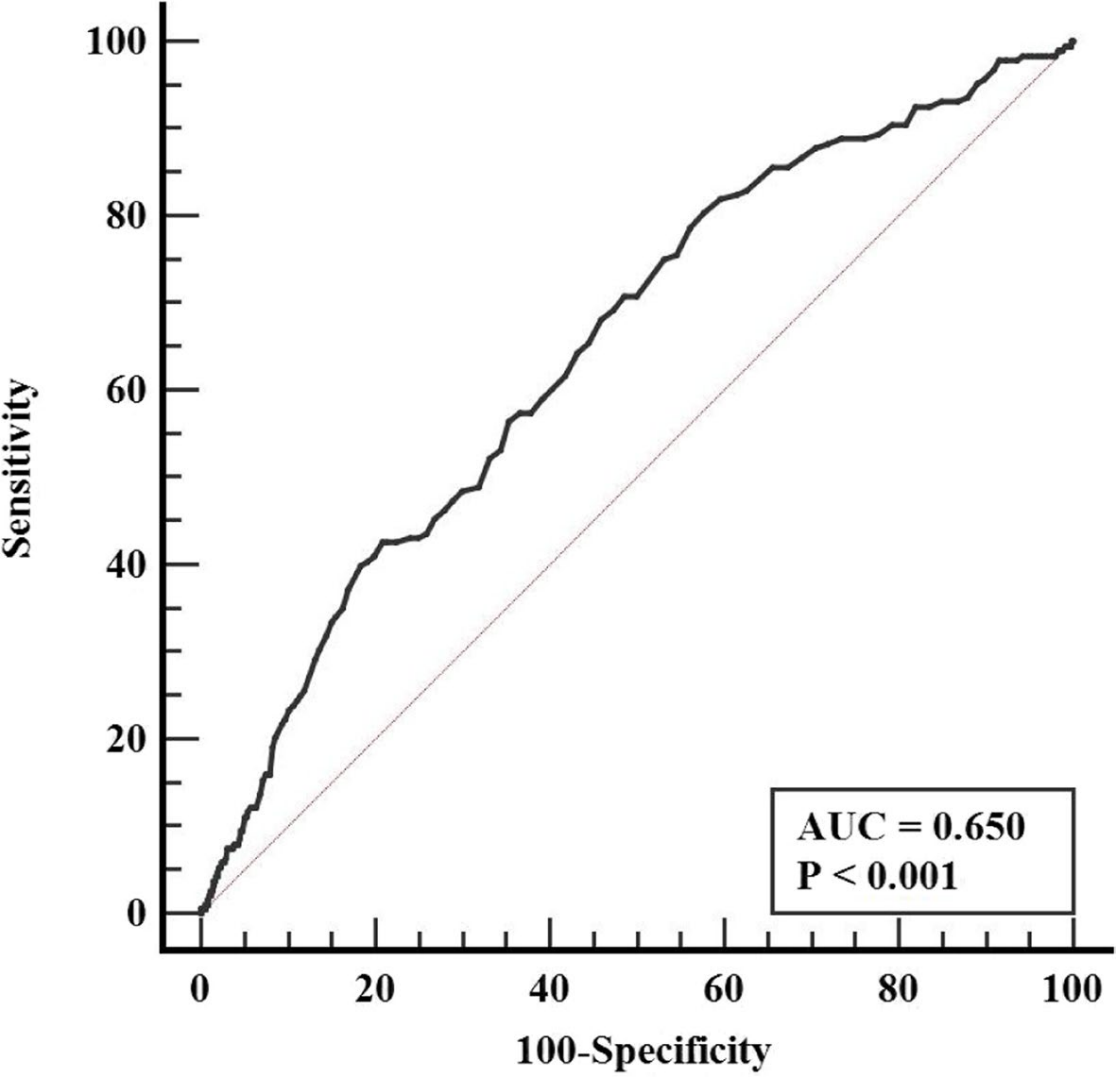
ROC curve evaluating predictive effect of ApoA1 for osteoporosis. ApoA1, apolipoprotein A1; AUC, area under the curve

As shown in Fig. 2, ROC analysis showed that ApoA1 could predict the occurrence of osteoporosis (AUC = 0.650, *P* <  0.001), and we divided all participants into two groups according to the optimal cutoff point of ApoA1 obtained by ROC: lower ApoA1 group (ApoA1 ≤ 1.36 g/L) and higher ApoA1 group (ApoA1 > 1.36 g/L). As shown in Table 1, participants with higher ApoA1 had higher age, higher proportion of women, non-Hispanic Black, daily smokers, hypertension, hypercholesterolemia, osteoporosis, and lower proportion of gout, and higher levels of BMI, SBP, TC, LDL-C, HDL-C, total calcium, and lower levels of TG, BUN, CR, albumin, uric acid, FPG, CRP, ALP, and 25(OH) D compared with participants who had lower ApoA1 (*P* <  0.05). As shown in Table 2, compared to participants without osteoporosis, participants with osteoporosis had higher age, higher proportion of women, non-Hispanic White, hypertension, diabetes, gout, use of hypotensive drugs, and use of hypoglycemic drugs, and had higher levels of SBP, TG, TC, LDL-C, HDL-C, ApoA1, ApoB, BUN, FPG, HbA1c, ALP, total calcium, and lower levels of albumin and uric acid (*P* <  0.05).

**Table 1:** Baseline characteristics stratified by the optimal cutoff point of ApoA1

	Total population (*n* = 7743)	Lower ApoA1 (*n* = 3359)	Higher ApoA1 (*n* = 4384)	*P* value
Age, years	49.20 ± 19.68	47.51 ± 19.71	50.49 ± 19.56	< 0.001
Sex, male, n (%)	3910 (50.50%)	2138 (63.60%)	1772 (40.40%)	< 0.001
Race, n (%)				< 0.001
Non-Hispanic White	3514 (45.40%)	1565 (46.60%)	1949 (44.50%)	
Non-Hispanic Black	1886 (24.40%)	623 (18.50%)	1263 (28.80%)	
Mexican-American	2100 (27.10%)	1066 (31.70%)	1034 (23.60%)	
Others	243 (3.10%)	105 (3.10%)	138 (3.10%)	
Smoking status, n (%)				< 0.001
Every day	3592 (46.40%)	1419 (42.30%)	2173 (49.60%)	
Some days	2044 (26.40%)	929 (27.70%)	1115 (25.40%)	
Not at all	2106 (27.20%)	1010 (30.10%)	1096 (25.00%)	
Comorbidities, n (%)				
Hypertension	2872 (37.10%)	1174 (35.00%)	1698 (38.70%)	< 0.001
Diabetes	1203 (15.50%)	511 (15.20%)	692 (15.80%)	0.213
Hypercholesterolemia	1240 (33.30%)	479 (30.90%)	761 (35.00%)	0.010
Gout	245 (3.20%)	123 (3.70%)	122 (2.80%)	0.047
Medication, n (%)				
Hypotensive drugs	1229 (15.90%)	520 (15.50%)	709 (16.20%)	0.702
Hypoglycemic drugs	443 (5.70%)	207 (6.20%)	236 (5.40%)	0.288
Cholesterol-lowering drugs	227 (8.20%)	107 (8.90%)	120 (7.60%)	0.225
BMI, kg/m^2^	26.77 ± 5.55	23.26 ± 3.90	24.51 ± 3.48	0.027
SBP, mmHg	126.12 ± 19.53	125.03 ± 18.57	126.95 ± 20.20	< 0.001
DBP, mmHg	74.08 ± 10.62	74.20 ± 10.54	73.99 ± 10.68	0.393
TG, mmol/L	1.29 (0.89, 1.93)	1.41 (0.97, 2.18)	1.19 (0.84, 1.74)	< 0.001
TC, mmol/L	5.37 ± 1.16	5.14 ± 1.11	5.56 ± 1.16	< 0.001
LDL-C, mmol/L	3.35 ± 1.00	3.31 ± 0.95	3.39 ± 1.04	0.030
HDL-C, mmol/L	1.33 ± 0.40	1.07 ± 0.24	1.54 ± 0.38	< 0.001
ApoB, g/L	1.07 ± 0.27	1.07 ± 0.26	1.06 ± 0.28	0.065
BUN, mmol/L	5.33 ± 2.26	5.40 ± 2.30	5.26 ± 2.22	0.008
CR, umol/L	96.49 ± 34.01	98.99 ± 36.10	94.55 ± 32.18	< 0.001
Albumin, g/L	42.34 ± 3.89	42.85 ± 3.98	41.94 ± 3.76	< 0.001
Uric acid, umol/L	318.37 ± 90.24	332.97 ± 89.18	307.06 ± 89.45	< 0.001
FPG, mmol/L	5.24 (4.89, 5.69)	5.27 (4.94, 5.72)	5.22 (4.85, 5.67)	< 0.001
HbA1c, %	5.52 ± 1.10	5.54 ± 1.12	5.50 ± 1.09	0.152
CRP, mg/dL	0.21 (0.21, 0.44)	0.21 (0.21, 0.44)	0.21 (0.21, 0.33)	0.002
ALP, U/L	84.66 ± 34.72	86.57 ± 29.72	83.18 ± 38.08	< 0.001
Total calcium, mmol/L	2.34 ± 0.11	2.33 ± 0.11	2.34 ± 0.11	0.005
25(OH) D, nmol/L	55.50 ± 20.15	56.10 ± 19.69	55.04 ± 20.48	0.023
Osteoporosis, n (%)	188 (2.40%)	40 (1.20%)	148 (3.40%)	< 0.001

Data were expressed as mean ± SD, median (interquartile range), or n (%). Lower ApoA1 ApoA1 ≤ 1.36 g/L, Higher ApoA1 ApoA1 > 1.36 g/L; *ApoA1* apolipoprotein A1; *BMI* body mass index; *SBP* systolic blood pressure; *DBP* diastolic blood pressure; *TG* triglycerides; *TC* total cholesterol; *LDL-C* low-density lipoprotein cholesterol; *HDL-C* high-density lipoprotein cholesterol; *ApoB* apolipoprotein B; *BUN* blood urea nitrogen; *CR* creatinine; *FPG* fasting plasma glucose; *HbA1c* hemoglobin A1c; *CRP* C-reactive protein; *ALP* alkaline phosphatase; *25(OH) D* 25-hydroxyvitamin D

**Table 2:** Baseline characteristics of participants stratified by the osteoporosis

	Non-osteoporosis (*n* = 7555)	Osteoporosis (*n* = 188)	*P* value
Age, years	48.76 ± 19.58	66.63 ± 15.18	< 0.001
Sex, male, n (%)	3887 (51.40%)	23 (12.20%)	< 0.001
Race, n (%)			< 0.001
Non-Hispanic White	3372 (44.60%)	142 (75.50%)	
Non-Hispanic Black	1868 (24.70%)	18 (9.60%)	
Mexican-American	2074 (27.50%)	26 (13.80%)	
Others	241 (3.20%)	2 (1.10%)	
Smoking status, n (%)			0.199
Every day	3493 (46.20%)	99 (52.70%)	
Some days	1998 (26.40%)	46 (24.50%)	
Not at all	2063 (27.30%)	43 (22.90%)	
Comorbidities, n (%)			
Hypertension	2762 (36.90%)	110 (58.50%)	< 0.001
Diabetes	1156 (15.30%)	47 (25.00%)	< 0.001
Hypercholesterolemia	1189 (33.10%)	51 (38.30%)	0.210
Gout	234 (3.10%)	11 (5.90%)	0.033
Medication, n (%)			
Hypotensive drugs	1169 (16.70%)	60 (33.90%)	< 0.001
Hypoglycemic drugs	424 (5.70%)	19 (10.10%)	0.010
Cholesterol-lowering drugs	214 (8.00%)	13 (13.50%)	0.051
BMI, kg/m^2^	26.77 ± 5.54	26.69 ± 6.08	0.846
SBP, mmHg	125.89 ± 19.50	135.84 ± 18.65	< 0.001
DBP, mmHg	74.08 ± 10.64	73.75 ± 9.54	0.679
TG, mmol/L	1.28 (0.88, 1.92)	1.52 (1.05, 2.07)	0.001
TC, mmol/L	5.36 ± 1.16	5.78 ± 1.09	< 0.001
LDL-C, mmol/L	3.35 ± 1.00	3.60 ± 0.97	0.035
HDL-C, mmol/L	1.33 ± 0.40	1.49 ± 0.44	< 0.001
ApoA1, g/L	1.44 ± 0.26	1.58 ± 0.29	< 0.001
ApoB, g/L	1.07 ± 0.27	1.14 ± 0.29	< 0.001
BUN, mmol/L	5.31 ± 2.26	5.97 ± 2.31	< 0.001
CR, umol/L	96.56 ± 34.22	93.50 ± 24.07	0.231
Albumin, g/L	42.38 ± 3.89	40.67 ± 3.13	< 0.001
Uric acid, umol/L	318.93 ± 90.08	295.73 ± 94.03	0.001
FPG, mmol/L	5.24 (4.89, 5.68)	5.38 (4.99, 5.89)	0.010
HbA1c, %	5.51 ± 1.09	5.74 ± 1.32	0.005
CRP, mg/dL	0.21 (0.21, 0.44)	0.21 (0.21, 0.44)	0.063
ALP, U/L	84.46 ± 34.58	92.58 ± 39.29	0.002
Total calcium, mmol/L	2.34 ± 0.11	2.36 ± 0.11	0.003
25(OH) D, nmol/L	55.52 ± 20.13	54.58 ± 21.05	0.545

Data were expressed as mean ± SD, median (interquartile range), or n (%)

*BMI* body mass index; *SBP* systolic blood pressure; *DBP* diastolic blood pressure; *TG* triglycerides; *TC* total cholesterol; *LDL-C* low-density lipoprotein cholesterol; *HDL-C* high-density lipoprotein cholesterol; *ApoA1* apolipoprotein A1; *ApoB* apolipoprotein B; *BUN* blood urea nitrogen; *CR* creatinine; *FPG* fasting plasma glucose; *HbA1c* hemoglobin A1c; *CRP* C-reactive protein; *ALP* alkaline phosphatase; *25(OH) D* 25-hydroxyvitamin D

### Association of ApoA1 with osteoporosis

As shown in the multivariate logistic regression analysis in Tables 3 and 4, when ApoA1 was used as a continuous variable, it was significantly associated with osteoporosis in all three adjusted models [Model 1, 2 and 3, OR (95% CI), *P* value: 2.219 (1.316, 3.739), 0.003; 2.381 (1.404, 4.038), 0.001; 2.289 (1.350, 3.881), 0.002; respectively]. And ApoA1, as a categorical variable, remained strongly associated with osteoporosis when adjusted for age, sex, race, hypertension, diabetes, gout, hypotensive drugs, hypoglycemic drugs, SBP, TC, LDL-C, HDL-C, ApoB, BUN, albumin, uric acid, HbA1c, ALP, and total calcium (Model 3, OR: 1.712, 95% CI: 1.183–2.478, *P* = 0.004). And whether ApoA1 was a continuous variable or a categorical variable, higher ApoA1 was consistently associated with a higher risk of osteoporosis after excluding individuals with gout and adjusting for confounding variables (All *P* <  0.01).

**Table 3:** Association of ApoA1 (categorical variable) with osteoporosis

		Model 1		Model 2		Model 3	
		OR (95% CI)	*P* value	OR (95% CI)	*P* value	OR (95% CI)	*P* value
With gout	Lower ApoA1	Ref	–	Ref	–	Ref	–
	Higher ApoA1^a^	1.695 (1.177, 2.443)	0.005	1.762 (1.220, 2.544)	0.003	1.712 (1.183, 2.478)	0.004
Without gout	Lower ApoA1	Ref	–	Ref	–	Ref	–
	Higher ApoA1^a^	1.764 (1.204, 2.584)	0.004	1.821 (1.240, 2.675)	0.002	1.816 (1.235, 2.668)	0.002

Model 1: adjusted for age and sex; Model 2: adjusted for age, sex, race, hypertension, diabetes, gout, hypotensive drugs and hypoglycemic drugs. Model 3: adjusted for variables included in Model 2 and SBP, TC, LDL-C, HDL-C, ApoB, BUN, albumin, uric acid, HbA1c, ALP and total calcium

*ApoA1* apolipoprotein A1; *SBP* systolic blood pressure; *TC* total cholesterol; *LDL-C* low-density lipoprotein cholesterol; *HDL-C* high-density lipoprotein cholesterol; *ApoB* apolipoprotein B; *BUN* blood urea nitrogen; *HbA1c* hemoglobin A1c; *ALP* alkaline phosphatase; *OR* odds ratio; *CI* confidence interval

^a^The OR was examined regarding lower ApoA1 as reference

**Table 4:** Association of ApoA1 (continuous variable) with osteoporosis

		Model 1		Model 2		Model 3	
		OR (95% CI)	*P* value	OR (95% CI)	*P* value	OR (95% CI)	*P* value
With gout	ApoA1^a^	2.219 (1.316, 3.739)	0.003	2.381 (1.404, 4.038)	0.001	2.289 (1.350, 3.881)	0.002
Without gout	ApoA1^a^	2.361 (1.387, 4.020)	0.002	2.511 (1.465, 4.301)	0.001	2.404 (1.401, 4.123)	0.001

Model 1: adjusted for age adjusted for age, sex, race, hypertension, diabetes, hypotensive and sex; Model 2: adjusted for age, sex, race, hypertension, diabetes, hypotensive drugs and hypoglycemic drugs. Model 3: adjusted for variables included in Model 2 and SBP, TC, LDL-C, HDL-C, ApoB, BUN, albumin, uric acid, HbA1c, ALP and total calcium

*ApoA1* apolipoprotein A1; *SBP* systolic blood pressure; *TC* total cholesterol; *LDL-C* low-density lipoprotein cholesterol; *HDL-C* high-density lipoprotein cholesterol; *ApoB* apolipoprotein B; *BUN* blood urea nitrogen; *HbA1c* hemoglobin A1c; *ALP* alkaline phosphatase; *OR* odds ratio; *CI* confidence interval

^a^The OR was examined by per 1-unit increase of ApoA1

### Subgroup analysis

When we conducted a subgroup analysis based on age and sex stratification (Table 5), we found that ApoA1 was closely associated with osteoporosis only among female participants [Model 1, 2 and 3, OR (95% CI), *P* value: 1.653 (1.103, 2.477), 0.015; 1.638 (1.092, 2.458), 0.017; 1.586 (1.053, 2.389), 0.027; respectively].

**Table 5:** Subgroup analysis of association of ApoA1 with osteoporosis

		Model 1		Model 2		Model 3	
		OR (95% CI)	*P* value	OR (95% CI)	*P* value	OR (95% CI)	*P* value
Age							
< 60 years	Lower ApoA1	Ref	–	Ref	–	Ref	–
	Higher ApoA1^a^	1.935 (0.997, 3.754)	0.051	1.910 (0.978, 3.731)	0.058	1.557 (0.717, 3.381)	0.263
≥ 60 years	Lower ApoA1	Ref	–	Ref	–	Ref	–
	Higher ApoA1^a^	1.590 (1.028, 2.459)	0.037	1.615 (1.038, 2.511)	0.034	1.460 (0.872, 2.442)	0.150
Sex							
Male	Lower ApoA1	Ref	–	Ref	–	Ref	–
	Higher ApoA1^a^	1.847 (0.797, 4.283)	0.153	1.850 (0.795, 4.303)	0.153	1.532 (0.544, 4.316)	0.420
Female	Lower ApoA1	Ref	–	Ref	–	Ref	–
	Higher ApoA1^a^	1.653 (1.103, 2.477)	0.015	1.638 (1.092, 2.458)	0.017	1.586 (1.053, 2.389)	0.027

Model 1: adjusted for age and sex; Model 2: adjusted for age, sex, race, hypertension, diabetes, gout, hypotensive drugs and hypoglycemic drugs. Model 3: adjusted for variables included in Model 2 and SBP, TC, LDL-C, HDL-C, ApoB, BUN, albumin, uric acid, HbA1c, ALP and total calcium. The model used in the subgroups analysis consisted of all covariates used in Model 1, 2 and 3 except for the variables that were used for stratification

*ApoA1* apolipoprotein A1; *SBP* systolic blood pressure; *TC* total cholesterol; *LDL-C* low-density lipoprotein cholesterol; *HDL-C* high-density lipoprotein cholesterol; *ApoB* apolipoprotein B; *BUN* blood urea nitrogen; *HbA1c* hemoglobin A1c; *ALP* alkaline phosphatase; *OR* odds ratio; *CI* confidence interval

^a^The OR was examined regarding lower ApoA1 as reference

## Discussion

In this cross-sectional study, we found for the first time a close association between ApoA1 and osteoporosis, and the significance of this association was not changed by confounding factors, suggesting that ApoA1 or lipid metabolism may be involved in the occurrence and development of osteoporosis.

Although both lipid metabolism and osteoporosis are strongly associated with the development of cardiovascular disease, there are few studies on the relationship between them and no consensus has been reached. In 2002, Yamaguchi et al. hypothesized that lipid dysregulation might be related to the pathogenesis of osteoporosis, and then confirmed their hypothesis that HDL-C and LDL-C levels were positively and inversely associated with the risk of osteoporosis, respectively, in a small study of only 214 postmenopausal Asian women [15]. Subsequently, a study by Cui et al. in 2005 showed that HDL-C was not significantly associated with the risk of osteoporosis at any site in both premenopausal and postmenopausal women [16]. In addition, Jeong et al. also found a positive association between HDL-C levels and risk of lumbar spine osteoporosis in postmenopausal women in a large cross-sectional study involving 10,402 women [17]. And Li et al. in 790 Chinese postmenopausal women also demonstrated that higher HDL-C levels were strongly associated with a higher probability of osteoporosis [18]. Besides, Wang et al. reaffirmed the above in an epidemiological study including 1791 Chinese participants aged ≥55 years that higher HDL-C levels were strongly associated with a higher risk of osteoporotic fracture, and this association remained stable in women [14]. Furthermore, Chen et al. demonstrated a causal positive association between HDL-C and the risk of osteoporotic fracture in older adults in a genome-wide association study [19], which further suggests that we should consider cardiovascular beneficial HDL-C as a risk factor for osteoporosis. However, in a meta-analysis and systematic review, Ghorabi et al. showed that lower HDL-C levels were associated with a higher risk of osteoporotic fractures [20]. Moreover, although Zhang et al. found a nonlinear association between HDL-C and bone mineral density in a study including 1116 women, HDL-C below 2.37 mmol/L was still negatively associated with bone mineral density in the lumbar spine [21]. And Kan et al. in a cross-sectional study also only found that higher TG and TC levels were associated with higher risk of osteoporosis, while no association between HDL-C and osteoporosis was found [22]. In summary, there is no consensus on the association between HDL-C and the risk of osteoporosis, and the above studies did not assess the association between ApoA1 and osteoporosis. Nevertheless, our study not only found HDL-C to be associated with osteoporosis, but also found that higher levels of ApoA1 were independently associated with a higher risk of osteoporosis. In addition, because gout has a great influence on osteoporosis, we further excluded participants with gout from multivariate regression analysis for sensitivity analysis. The results showed that the relationship between ApoA1 and osteoporosis remained stable after excluding individuals with gout. Unfortunately, because our study was the first report of an association between ApoA1 and osteoporosis, we were unable to perform a comparative analysis with similar studies. Additionally, the potential pathological mechanism between ApoA1 and osteoporosis is unknown, and more basic and clinical studies are needed to further explore the mystery.

Although we found for the first time that higher levels of ApoA1, which has cardiovascular benefits, were strongly associated with a higher risk of osteoporosis, there were still some limitations to our study. First, the causal relationship between ApoA1 and osteoporosis could not be determined because of the limitations of the observational study. Second, because osteoporosis was diagnosed in this study based on participants’ self-reported medical history, the results may have been biased. Third, because all participants were from the US population only, the relationship between ApoA1 and osteoporosis in other countries is still unknown. Fourth, because this study was an epidemiological study without evidence from cellular and animal experiments to support it, the mechanisms involved are still unknown. Fifth, this study participated only 23 male subjects with osteoporosis, so there is a possibility of statistical under-detection. Sixth, not only ApoA1 varies from race to race, but also lipoprotein a varies from race to race, so we adjusted the confounding factor of race in the study and found that the main results were still stable. Although some genetic variants of apolipoprotein E may lead to higher prevalence in some races, there is no clear mechanism to explain this finding. Finally, due to the restrictive nature of the data, we may not be able to include all confounding factors. Nevertheless, this study was also very relevant to support the argument that lipid metabolism is involved in bone metabolism.

## Conclusions

In this study, we found for the first time that higher ApoA1 levels were strongly associated with a higher risk of osteoporosis, which not only fills this knowledge gap, but also suggests that lipids with cardiovascular benefits may be detrimental to osteoporosis, and moreover, provides a reference and theoretical basis for the development of treatment strategies appropriate for specific populations. In addition, these results also suggest that there may be many common pathways in the pathogenesis of metabolism-related diseases, such as lipid metabolism, osteoporosis and cardiovascular disease. Therefore, in the management of these diseases, we should consider as many factors as possible to develop individual-specific diagnosis and treatment programs, so that we can more accurately prevent and treat metabolic-related diseases.

## Data Availability

All data and materials can be downloaded from a public database (https://www.cdc.gov/nchs/nhanes/index.htm).
